# How integration of refugees into national health systems became a global priority: a qualitative policy analysis

**DOI:** 10.1186/s13031-024-00587-4

**Published:** 2024-04-15

**Authors:** Shatha Elnakib, Caitlin Jackson, Ummekulsoom Lalani, Yusra Ribhi Shawar, Sara Bennett

**Affiliations:** 1 Johns Hopkins Bloomberg School of Public Health, Baltimore, MD USA; 2 Paul H. Nitze School of Advanced International Studies, Washington, DC USA

**Keywords:** Refugee Integration, Health systems, Policy analysis, Shiffman and Smith Policy Framework

## Abstract

**Background:**

Despite a long history of political discourse around refugee integration, it wasn’t until 2016 that this issue emerged as a global political priority. Limited research has examined the evolution of policies of global actors around health service provision to refugees and how refugee integration into health systems came onto the global agenda. This study seeks to fill this gap.

**Methods:**

Drawing on a document review of 20 peer-reviewed articles, 46 global policies and reports, and 18 semi-structured interviews with actors representing various bilateral, multilateral and non-governmental organizations involved with refugee health policy and funding, we analyze factors that have shaped the global policy priority of integration. We use the Shiffman and Smith Policy Framework on determinants of political priority to organize our findings.

**Results:**

Several important factors generated global priority for refugee integration into national health systems. Employing the above-mentioned framework, actor power increased due to network expansion through collaborations between humanitarian and development actors. Ideas took hold through the framing of integration as a human rights and responsibility sharing. While political context was influenced through several global movements, it was ultimately the influx of Syrian refugees into Europe and the increasing securitization of the refugee crisis that led to key policies, and critically, global funding to support integration within refugee hosting nations. Finally, issue characteristics, namely the magnitude of the global refugee crisis, its protractedness and the increasing urbanicity of refugee inflows, led integration to emerge as a manageable solution.

**Conclusion:**

The past decade has seen a substantial reframing of refugee integration, along with increased financing sources and increased collaboration, explains this shift towards their integration into health systems. However, despite the emergence of integration as a global political priority, the extent to which efforts around integration have translated into action at the national level remains uncertain.

**Supplementary Information:**

The online version contains supplementary material available at 10.1186/s13031-024-00587-4.

## Introduction

The acceleration of forced displacement has emerged as one of the greatest challenges of the twenty first century. By mid-year 2022, over 100 million people were forcibly displaced, an unprecedented number since the second world war [1]. Historically, refugees resided in camps operated by UNHCR with the aspiration that one of three durable solutions – repatriation, integration into host country, or third country resettlement – would eventually become possible. The question of how to organize health service delivery to refugees in the interim was extensively debated. UNHCR’s longstanding approach to health delivery was a ‘care and maintenance’ approach, where care for refugees was provided through dedicated clinics run primarily by international non-governmental organizations (INGOs) [2]. These services, provided through hospitals and clinics, were mostly set up inside camps, often at a higher standard of care than pre-existing health systems in the host country [3]. This approach, however, had many shortcomings. It bred long-term dependence, ignored many refugees who resided outside of camp settings, and became increasingly unsustainable as contemporary crises became more protracted.

In 2016 and 2017, there was a paradigm shift away from ‘care and maintenance’ toward integration and inclusion of refugees into host country national health systems. Foreshadowing this shift, in 2016, the founder of the World Bank’s Global Program on Forced Displacement noted: “Work on forced displacement is at a crucial moment, a tipping point. It is the right time to consolidate the paradigm shift towards full global recognition that the challenge of forced displacement is an integral part of the development agenda” [4]. In the wake of the Mediterranean refugee crisis, the 2016 World Humanitarian Summit facilitated conversations around drastic reform in humanitarian response called the Grand Bargain [5]. At the summit, the World Bank committed to stepping up financing of refugees and host governments [6]. The conference also set the stage for the 2016 New York Declaration for Refugees and Migrants, which was unanimously adopted by the United Nations General Assembly [7]. The explicit position of the New York Declaration was to address the health needs of refugees by integrating them into the national health care and social protection systems of the countries that host them. At the same time, the policy calls on the international community to support host countries in this process to share the responsibility of addressing the unprecedented level of human mobility [7]. Described by UN High Commissioner for Refugees Filippo Grandi as a “political commitment of unprecedented force and resonance,” the Declaration “fills what has been a perennial gap in the international protection system – that of truly sharing responsibility for refugees” [8].

The New York Declaration for Refugees and Migrants [7] laid the foundation for the UNHCR led Comprehensive Refugee Response Framework (CRRF) [9] and Global Compact on Refugees (GCR) [10]. These global policies advocated for integration of refugees into national host systems, including national health systems. The 2018 affirmation of the GCR by States clearly demonstrated sustained support for and prioritization of refugee integration into health systems (along with broader inclusion into national systems).

However, the call to support refugees by integrating them into host country systems is not new. In fact, efforts and calls for the adoption of a developmental approach to forced displacement can be traced back as early as the 1960s, and in the late 1990s and early 2000s, several policies and programs were developed with a focus on promoting self-reliance, including the 1999 Self Reliance Strategy [11] introduced by UNHCR in Uganda which was one of the first in-country experiences with integration. These policies however did not translate into global policy and the ‘maintenance and care’ approach prevailed. Despite a long history of political discourse and experimentation, it wasn’t until 2016 that integration finally got on the global agenda [1, 2]. Drawing on the *Shiffman and Smith framework on determinants of political priority for global initiatives* [12], we aimed to examine factors that explain the recent emergence and growth of policy attention for the integration of refugees into health systems.

## Methods

### Policy framework

We used Shiffman and Smith’s policy framework (Table 1) to analyze the factors that contributed to how refugee integration into health systems became a global political priority. The framework includes 11 factors determining political prioritization that are grouped into 4 themes: (1) actor power, (2) ideas, (3) political contexts, and (4) issue characteristics. Shiffman and Smith define global priority as “the degree to which international and national political leaders actively give attention to an issue, and back up that attention with the provision of financial, technical and human resources that are commensurate with the severity of the issue” [12]. While other frameworks for explaining political priority exist [13], the Shiffman and Smith framework is focused at global, rather than national, agenda-setting. The framework has been widely applied in global public health [14–16] and the factors identified in this framework closely reflected the themes that emerged from our data.

**Table 1:** Shiffman and Smith framework on factors shaping political priority for global initiatives

Theme	Description	Factors
Actor Power	The strength of the individuals and organizations concerned with the issue	1. Policy community Cohesion 2. Leadership 3. Guiding Institutions 4. Civil Society mobilization
Ideas	The ways in which those involved with the issue understand and portray it	5. Internal frame 6. External frame
Political Contexts	The nature of the political climate in which actors operate	7. Policy windows 8. Global governance structure
Issue Characteristics	Features of the issue itself, including the extent to which a solution exists to the problem.	9. Credible indicators 10. Severity 11. Effective interventions

Reproduced from Shiffman and Smith (2007) [12]

### Study design

We conducted a case study of how political priority was achieved for global policies on integrating refugees into health systems. Our analysis was based on interviews with 18 key informants (Table 2) and a systematic search for relevant literature and documents, of which 66 documents were included in the analysis. We thematically analyzed the literature and interviews through triangulation of data.

### Document review

We collected data from several data sources, including 20 peer-reviewed and 46 grey literature documents. The search included retrieving peer-reviewed articles through a comprehensive search of the following databases: Google Scholar, PubMed, and Embase. The following search terms were used: “inclusion”, “integration”, “integrated delivery of health care”, “integrated health care system*”, “integrated delivery systems” with a focus on refugees and displaced persons. The search was restricted to articles in English and those published before September 2022. The two first authors conducted title, abstract and full-text screening on the retrieved articles.

This was complemented by a grey literature search with archival research in which we collated and reviewed organizational reports, conference documents and meeting minutes and briefs, policy statements, media reports, press releases and public statements, legislations including laws, decrees, and conventions. The grey literature was collated by searching 25 organizational sites that work with refugee populations. Seminal legislation and global policies that focused on refugees and refugee service delivery, and organizational policies and decisions that described health system responses to refugee crises were included in this review. Literature that exclusively focused on migrants and did not specifically reference refugee populations was excluded. Additionally, grey literature identified through the qualitative interviews was also reviewed against the inclusion and exclusion criteria above.

**Fig. 1:**
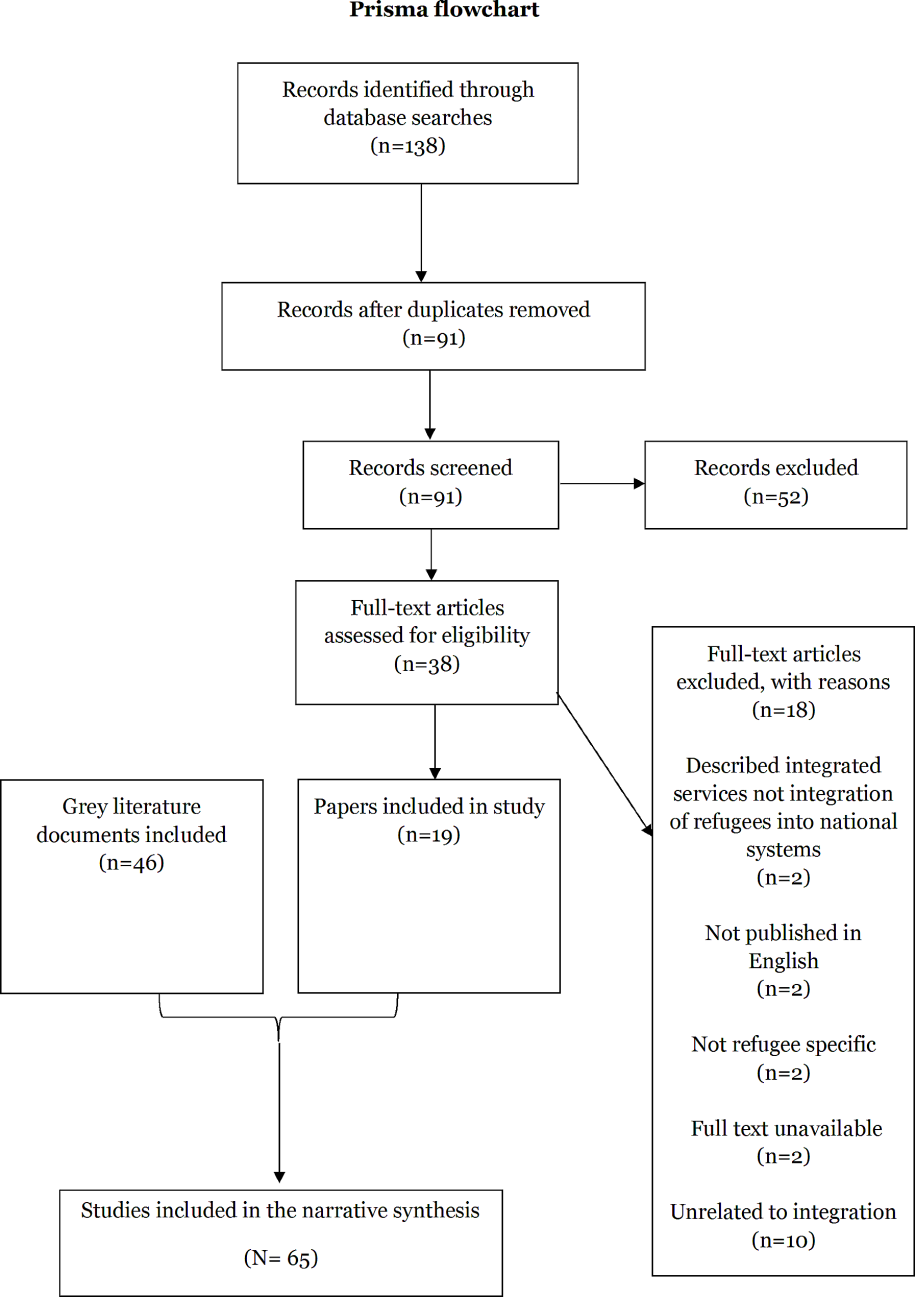
Flow diagram for peer-reviewed literature review (until September 2022)

Of the 138 peer reviewed documents identified and deduplicated, 91 were screened by title and abstract (Fig. 1). 52 documents were excluded. The 38 remaining articles underwent full text review and 18 were ineligible for inclusion. Ultimately, 19 peer reviewed publications were included in the study. 64 grey literature documents were identified and underwent full text review. 18 were excluded. A total of 46 grey literature documents were included in the study.

The grey and peer reviewed literature were primarily published between 2016 and 2022. Most grey literature was published by UN agencies (*n* = 21), followed by the World Bank (*n* = 7). The grey literature primarily comprised annual reports and policy briefs. The focus of most peer reviewed literature was on LMICs (*n* = 12), followed by European countries (*n* = 7). The studies were primarily qualitative, literature reviews, or normative papers focused on policy review and analysis.

Documents that were eligible for inclusion were reviewed by the first and second author, and data was extracted by the second author using a charting form sheet developed by both authors. Data entry was done in Excel and the extraction included author name, study title, year and type of publication, geographic focus, as well as information about the following themes: meaning of integration, historical evolution of discourse around integration, rationale and enablers of integration, barriers to integration, successful and unsuccessful examples, funding, and collaboration.

### Key informant interviews

We conducted 18 interviews with 24 individuals centrally involved in refugee health policy and funding at the global level. While the majority of interviews were with one individual, four interviews were with 2–3 individuals who worked in the same organization. The initial group was purposively selected and was expanded upon through snowball sampling in which we asked participants to identify additional participants who can provide insight into the research question. Key informants were also identified through the research team’s knowledge of the field. We contacted 42 potential key informants and conducted 18 interviews (43% response rate). Initial interviews were conducted between September and October 2020, and after carefully reviewing these, a second round of interviews was conducted between November and December 2022 with additional respondents to try to fill in particular gaps in perspective. Table 2 lists the informants’ institutional affiliations. We stopped conducting more interviews when we reached the point of theoretical saturation [17].

**Table 2:** Profile of participants in key informant interviews

Type of organization/Institution	Sample size
International non-governmental organization	1
UN agencies	8
World Bank	3
Bilateral agency/ Government organization	3
Academia	2
Development Firm	1

Informants were contacted through a standardized email and asked to participate in a phone or online interview via Microsoft Teams (Microsoft Corporation; Redmond, WA, USA) or Zoom (Zoom Video Communications; San Jose, CA, USA). Interviews were semi-structured (See Supplementary File 1 for the interview guide). They were conducted in English and lasted for approximately an hour and were recorded, and transcribed verbatim. The first, second and third authors conducted the interviews.

### Qualitative analysis

We conducted thematic analysis of the interview data and narrative synthesis of the retrieved literature. We initially used a codebook that included deductive codes based on the research questions, but then allowed new codes to emerge inductively. The first and second authors coded the data on Dedoose 9.0.62, a data management software [18]. The first author developed detailed memos and held regular debriefs with the second author to ensure consistency of coding and reflect on emerging findings.

### Ethical approval

The Johns Hopkins University Institutional Review Board in Baltimore, MD, USA reviewed the protocol and determined that it did not qualify as human subjects research, thereby granting it an exemption.

## Results

### Global evolution of refugee health integration policy

**Fig. 2:**
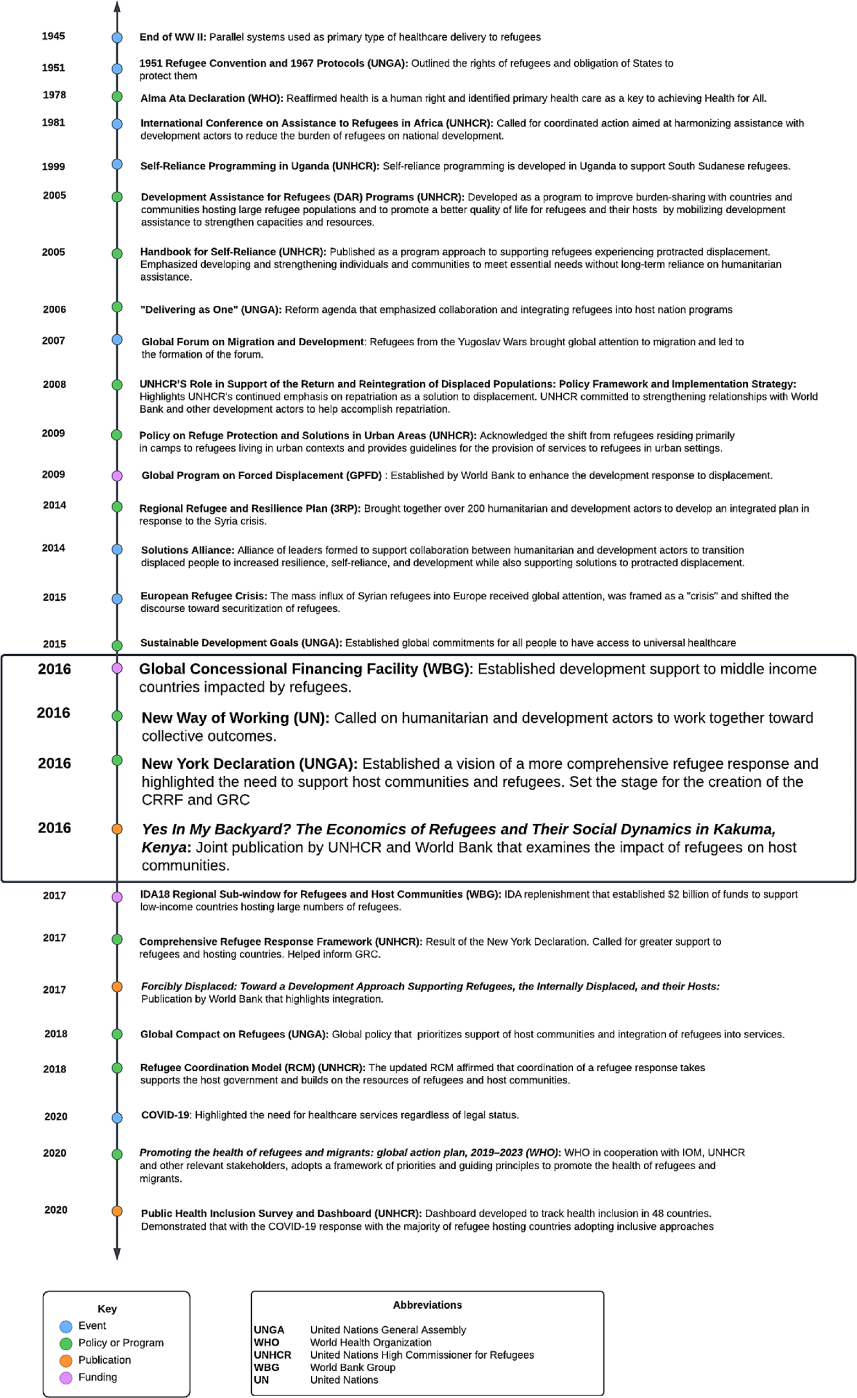
Abridged Timeline of events leading to the adoption of policies that encourage integration of refugees into health systems

We summarized our findings by creating a timeline of the evolution of global policies and key events around health service delivery to refugee populations. (Fig. 2: Abridged Timeline of Key Events Surrounding Integration; See Supplementary File 2 for the full version).

At the end of WWII, displacement challenges received global attention. At this time, parallel health systems were established to temporarily support refugees while awaiting repatriation. The first key global policy of significance was the 1951 UN Convention Relating to the Status of Refugees [19]. Refugees were the responsibility of host governments. When needed and invited by hosting governments, humanitarian actors stepped in to provide aid. UNHCR oversaw refugee health with the support of implementing humanitarian organizations that provided services for refugees. There was a clear demarcation between the work of humanitarians in supporting refugees and the work of development actors, who were frequently active in refugee hosting countries. As early as 1967, Prince Sadruddin Aga Khan, then High Commissioner for Refugees, discussed the need to address refugees through a development approach, where host communities are supported as well. In an address to the United Nations General Assembly (UNGA), the High Commissioner went so far as to say that the response to the refugee situation in Africa could not be effective unless multilateral development aid was coordinated with the humanitarian refugee response. He argued that development assistance to strengthen refugee hosting regions would optimize resource use and avoid waste [20]. Another attempt to shift the paradigm toward integration took place at the 1981 International Conference on Assistance to Refugees in Africa also hosted by UNGA. Leaders called for coordinated action aimed at harmonizing humanitarian assistance with development actors to reduce the burden of refugees on national development [21]. Again, calls for integration failed to galvanize collective action.

Several major events of displacement at the end of the 20th century directed the spotlight towards the issue of refugee assistance and how best to organize services in the wake of mass population movement. By the end of 1979, 400,000 Afghans had fled to Pakistan [22]. The Berlin Wall fell in 1989, bringing the issue into public view as East German refugees freely crossed the border [23]. In the late 80s over 25,000 Sudanese youth fled to Ethiopia and Kenya [18, 24]. There was also mass influx of refugees from the Yugoslav wars in the early 1990s [23]. Repatriation, the preferred durable solution of the era, became increasingly unfeasible due to the volume and protractedness of these displacement events.

Against a backdrop of mass displacement events and increasing funding shortfalls, new policies and programs began to develop in the late 90s and early 2000s with a focus on self-reliance for refugees. Of note is the 1999 Self Reliance Strategy introduced by UNHCR and the government of Uganda [25]. In 2001, UNHCR hosted a panel discussion on protracted refugee situations in Africa to develop better solutions for meeting the needs of refugees [25]. It was becoming clear that the traditional durable solutions for refugees, such as voluntary repatriation or third country resettlement, were not viable, and a published discussion paper suggested examining local integration [26]. It further suggested pursuing refugee self-reliance to support the needs of refugees in exile [26, 19]. However, the move towards integration did not take off, and the Self Reliance Strategy, which was one of the first experiments with integration, failed in part due to the complexities of the host government political environment, hesitation of host governments to call attention to refugees residing outside of camps, and the lack of guidance on how to implement integration [25]. In 2005 UNHCR published the Handbook for Self-Reliance [27], further reinforcing UNHCR’s shift in focus to self-reliance by providing an operational tool for field-based staff to implement self-reliance strategies. The handbook defined self-reliance in the context of programming as “developing and strengthening livelihoods of persons of concern and reducing their vulnerability and long-term reliance on humanitarian/external assistance” [27]. UNHCR also promoted Development Assistance for Refugees (2005), which recognized the growing concerns around finding durable solutions for refugees and proposed new concepts and programs from a development approach [28].

In 2009, the UNHCR Policy on Refugee Protection and Solutions in Urban areas was adopted, bringing broader recognition to the shift in refugee concentration from camps to urban settings [29]. UNHCR also began tracking accommodation type in 2010 to substantiante the reality that refugees reside among host populations [30]. Additionally, UNHCR began to explore funding mechanisms to further operationalize integration. In an attempt to support integration of Afghan refugees into the Iranian healthsystem, UNHCR released a guidance note in 2012 to propose possible health insurance schemes to allow refugees access to essential PHC and emergency services [31]. During this period, the concept of collaboration between development and humanitarian actors to strengthen host communities was underscored in the 2014 Regional Refugee and Resilience Plan [32], which brought together 270 UN agencies, INGOS, and NGOs and was jointly implemented by UNHCR and UNDP. The Solutions Alliance was launched in 2014, bringing together a group of donor and host governments, UN agencies, multilateral financial institutions, civil society organizations, international NGOs, private sector, and academic organizations dedicated to finding innovative solutions for protracted displacement [33]. In 2015, the Sustainable Development Goals (SDG) were adopted by UNGA, calling for universal health coverage for all individuals [34]. While the original iteration of the SDGs did not include any reference to refugees, reflecting the normative exclusion of refugees from discourse on sustainable development, advocacy on the part of humanitarian agencies spearheaded by UNHCR resulted in the inclusion of an indicator on refugees [35].

It was in this global environment, in 2015, that Europe experienced a significant increase in migration, known globally as the Mediterranean refugee crisis and linked to the influx of Syrian refugees, but also refugees from other countries such as Afghanistan, Eritrea, Iraq, Nigeria and Pakistan. This event served as a catalyst to change policy and mobilize stakeholders to forge new solutions for refugees. The World Bank, UN, and Islamic Development Bank met in October 2015 to discuss financing of the crisis, which in 2016 formally became the Global Concessional Financing Facility (GCFF), a financing mechanism to support countries hosting large numbers of refugees [36].

The pivotal 2016 Grand Bargain at the World Humanitarian Summit occurred in the wake of the Mediterranean refugee crisis and resulted in 65 signatories consisting of large donors and humanitarian organizations committing to improving effectiveness and efficiency in humanitarian action [5]. Of particular significance, the Summit resulted in the World Bank’s commitment to finance refugees and host governments [6] and led to the adoption of the 2016 New York Declaration for Refugees and Migrants [7]. The Humanitarian-Development Nexus and “New Way of Working” were also established in 2016, setting the stage for increased collaboration between humanitarians and development actors, particularly on refugee issues [37].

The 2016 commitments at the World Humanitarian Summit and in the New York Declaration for Refugees and Migrants produced tangible results. The CRRF [9] was a process used to develop the eventual GCR [10] that emphasized shared responsibility to support refugees and hosts. Underpinning this concept was the idea of integration. In discussing the CRRF, UNHCR notes that “allowing refugees to benefit from national services and integrating them into national development plans is essential for both refugees and the communities hosting them, and is consistent with the pledge to ‘leave no one behind’ in the 2030 Agenda for Sustainable Development” [9]. The World Bank’s commitments materialized with the establishment of the IDA18 Regional Sub-Window for Refugees and Host Communities, which provided an unprecedented $2 billion of dedicated funding to support low income countries that host refugees [38]. Furthermore, tworeports brought attention to integration. *Yes In My Backyard? The Economics of Refugees and Their Social Dynamics in Kakuma, Kenya* (2016) was jointly published by UNHCR and World Bank [39]. *Forcibly Displaced: Toward a Development Approach Supporting Refugees, the Internally Displaced, and their Hosts (2017)* was published by World Bank with a forward by UNHCR [40].

Finally, after 2 years of consultation and development, the Global Compact on Refugees was affirmed in 2018. The compact has four main objectives, namely to: (1) ease pressures on host countries; (2) enhance refugee self-reliance; (3) expand access to third country solutions; (4) support conditions in countries of origin for return in safety and dignity. The Compact specifically addresses health integration in Sect. 2.3 Health [10]. The 2018 affirmation of the GCR by States clearly demonstrated the support for and prioritization of refugee integration into health systems.

It was in this ideal environment for health integration that the SARS-CoV-2 pandemic tested even the most advanced health systems. While the pandemic could have soured political sentiment toward health integration, it lent support to the policy and provided clear opportunities for international support of national health systems to benefit both nationals and refugees. Many countries provided COVID-19 vaccines to refugees as part of their pandemic response, a clear shift from previous epidemic responses [41, 42].

As illustrated by the timeline, a long history of political discourse around integration predated the 2016 events that saw the issue emerge as a political priority and prevailing paradigm. Why and how the issue acquired global priority status is investigated in the next section using the Shiffman and Smith policy framework.

### Actor power: Network expansion and entrance of the World Bank onto the humanitarian scene

Network expansion through increased collaborations between humanitarian and development actors, as well as the critical engagement of World Bank, played a significant role in shifting the discourse on refugee integration into national health systems. As the custodian of the international refugee response regime, UNHCR has historically been at the helm of conversations around integration and while bridging the humanitarian development nexus was the subject of debates for decades, convergence between the humanitarian and development agendas and collaboration between actors had not materialized. Key informants credited the entrance of the World Bank onto the humanitarian scene in 2016 as a pivotal moment that translated rhetoric on a development approach to forced displacement and promoted social, economic and health integration into action. With the World Bank scaling up its involvement with refugees, including health integration, other international financial institutions (IFIs) such as the Islamic and Asian Development Bank followed suit. As one informant noted:

*The big game changer is the appearance of the World Bank on the scene where, at some point, the World Bank decided that they will do what they call risky investments and healthcare services, where it’s by no means sure that it will be a success*.

*Key Informant Interview, UN Agency*.

Collaboration between the World Bank and UNCHR was evidenced by the multiple joint missions that the two actors undertook. In 2017, in preparation for the World Bank International Development Assistance (IDA) 2018–2020 refugee and host community replenishment sub-window, the World Bank and UNHCR undertook 11 joint missions. The sub-window underpins a development approach to forced displacement and promotes the integration of refugees into national host systems. It presented a paradigm shift in that it offered a large amount of concessional loans which have zero or very negligible interest rates and long repayment periods stretching over 30 to 40 years UNHCR was additionally invited to serve as an observer to the Steering Committee Meetings of the Global Concessional Financing Facility (GCFF), a newly established fund housed at the Bank that supported countries responding to refugee crises.

Speaking about the evolution of the World Bank’s role in humanitarian response in 2016 and the expansion in the network of advocates working to move this agenda forward, Niels Harild, the founder of the World Bank’s Global Program on Forced Displacement stated:

“*No one actor can do this alone. The World Bank Group needs a strong partnership approach on this agenda, working closely with bilaterals, the UN - particularly UNHCR, NGOs, research institutions, the private sector and most importantly affected Governments*” [4].

*Niels Harild, World Bank Group*.

Because integration in health systems was situated in broader discourse around integration of refugees into social services more generally, the network of actors that came together was not exclusive to the health sector. A diverse network of advocates from education, health and labor coalesced to advance the integration agenda. The Solutions Alliance illustrated the diversity of actors involved in this effort. The Alliance brought together actors across different sectors and specialties, drawing on the expertise of academics, civil society organizations, UN agencies, international NGOs, multilateral agencies, and host and donor governments. The Alliance closed on June 17, 2017 after it was decided that “refugee self-reliance and resilience among affected communities has now gained broad political legitimacy” [43].

### Ideas: framing of integration in terms of human rights and responsibility sharing

Integration advocates were able to strategically tailor the language surrounding integration to resonate with specific audiences and linked it to the broader discourse surrounding ‘responsibility sharing’ and human rights. Despite heterogeneity in terms used by global refugee policy actors to describe the process of mainstreaming refugees into host health systems, there was consensus in understandings of the concept of integration and agreement that the preferred service delivery modality in humanitarian settings has shifted from vertical programs to integrated services delivered through existing host country health systems. There were calculated and deliberate reasons for why respondents chose different terms to describe the same phenomenon. As noted by one respondent, “*we have to be careful with that term (integration). So that’s why we will be saying integrating into service delivery*” (Key Informant Interview, UN Agency).

For many national policymakers, the term “integration’ was not associated with integration into health systems, but rather on policies focused on legal status. The term was often associated with naturalization or policy that allows refugees to become full and permanent members of society. Meanwhile, “inclusion” was typically used by policymakers to describe the inclusion of refugees in existing policies and systems. As an example, policymakers were familiar with policies advocating to include refugees in the national education system or policies to include refugees under existing national protection laws. As such, many respondents suggested national governments resisted the integration agenda because of concerns it would lead to the permanent naturalization of refugees. In place of integration, respondents used terms such as inclusion, self-reliance, and interim integration to describe the same policy concept and assuage concerns around the “permanent” presence of refugees.

Clear themes emerged in how actors framed integration to generate political priority for the issue and overcome pushback by governments. Our key informants noted that local decision-makers had strong fears that integration was a way to shift the buden of hosting onto local governments and would result in conditions that welcome refugees’ prolonged presence in their countries. Integration was deliberately and strategically framed by UNHCR in terms of ‘responsibility sharing,’ with the role of the global community in jointly addressing problems associated with displacement underscored in the discourse around integration. While usually used by the international refugee regime to discuss durable solutions for refugees, ‘responsibility sharing’ was successfully linked by actors to health integration policy, most notably expressed in the Global Compact on Refugees [10]. One informant noted:

*We’ve pitched it in a more sort of hopeful manner of international responsibility sharing. And that’s been the discourse…. Used to be called burden sharing. Now it’s responsibility sharing. And so we pitched it in that sense.*

*Key Informant Interview, UN Agency*.

Moreover, the framing of integration as more than a public health issue, and instead as a human right and the invocation of rights-based language helped advance the agenda. Actors were able to align and scaffold integration within existing international norms and global calls for action that positioned refugee access to healthcare as a human right. The literature credits the 1951 Refugee Convention [19] and 1967 Protocol relating to the Status of Refugees [44] as setting the foundation for refugees’ right to health, education, and workforce participation at the same level as residents (PR01, GL32). While dating back to the 1950s, this language was revitalized by proponents of integration. In addition to bolstering their case that integration was a human right, international treaties and conventions gave actors a mechanism for holding countries accountable as signatories of the Refugee Convention.

### Political context: the role of the Mediterranean refugee crisis and other concurrent global movements

Proponents of integration successfully connected integration with other global movements and leveraged an open policy window—the Mediterranean refugee crisis — to move integration policy forward. With momentum generated by the 2015 SDGs for universal health coverage, advocates pitched integration as an opportunity to upgrade national health systems and move towards universal healthcare coverage for all, including refugees and asylum seekers. At the same time, proponents were able to anchor integration policy in the broader movement within the UN system to bridge the humanitarian development nexus as the future of sustainable aid. The foundation of the 2006 Delivering as One [45] movement followed by the 2016 New Way of Working and Humanitarian Development Nexus [37] served as platform. As noted by an informant,

Interesting might be to add on, is not directly the policy frame, but I think the whole debate and discourse on the humanitarian development nexus discussion and the rationale behind that is for sure a big driver, a big push for the topic of inclusion.

*Key Informant Interview, European Bilateral*.

Since the integration paradigm largely rested on collaboration between humanitarian and development sectors, this emerging collaborative governance structure was pivotal in creating the political context for integration and in bringing actors together for collective action.

While synergies with global movements like universal health coverage and Humanitarian Development Nexus helped advance the integration agenda, it was ultimately the Mediterranean refugee crisis and specifically inflows of Syrian refugees into Europe that galvanized action on integration. The geopolitical significance of the Syria crisis and ensuing securitization of asylum in Europe – namely the imposition of restrictions on refugee movement into European Union countries – were portrayed as opening a policy window for integration. One respondent specifically noted the role of the Syrian refugee influx in bringing the World Bank on as a key actor in refugee integration policy.

*And here I’m thinking particularly of the World Bank. And we’ve talked for many, many years about the World Bank getting involved in the refugee situation so history goes back to the early 1980s. It never really happened until maybe the last four or five years it’s began to happen on a significantly greater scale. And again that’s been largely prompted by the Syrian refugee emergency*.

*Key Informant Interview, Academia*.

Many documents and policies adopted shortly after the Mediterranean refugee crisis (such as the New York Declaration (2016), the Comprehensive Refugee Response Framework (2017) and the Global Compact on Refugees (2018)) have been key in promoting integration of refugees into health systems.

Policies were ultimately strengthened by the emergence of corresponding funding instruments that helped integration become a political reality. The 2016 Global Concessional Financing Facility, established one year after the influx of refugees in Europe, established development support for countries impacted by refugees and was the precursor to the IDA Regional Sub-window for Refugees and Host Communities. Respondents described these funding mechanisms as crucial to the translation of integration policy into action.

In summary, proponents of integration successfully capitalized on global policy windows to elevate priority for integration, including the movement for universal health coverage, and the movement to bridge the humanitarian development nexus. The key catalyst however was presented as the Mediterranean refugee crisis which raised the profile of the issue, and prompted integration polices to emerge with the corresponding funding instruments needed to make integration a reality.

### Issue characteristics: leveraging data and indicators to make the case for integrtaion

Several aspects of the issue generated the momentum needed for this ideological shift in thinking around refugee response. This included the unprecedented scale of mass displacement occurring in the last two decades, the protracted nature of contemporary refugee crises, and the increasingly urbanized nature of refugee concentrations. UNHCR produced data and projections on the scale of displacement, which revealed that the world was witnessing the highest rates of population movement since the Second World War [46]. Analysis done by the World Bank empirically showed that the average duration in displacement was increasing, hovering around 10 years [47]. The increasing urbanization of refugees highlighted the limits of parallel service delivery and ‘care and maintenance’ approaches that failed to meet the needs of people residing outside of the confines of refugee camps. These realities occurred against a backdrop of budgetary shortfalls and constraints. The scale of displacement was simply outstripping available funding.

Key informants credited the emergence of empirical data and economic analysis of the impact of protracted refugee crises in part to the involvement of the World Bank. Analyses published in seminal reports prepared jointly by the World Bank and UNHCR, such as the “Forcibly Displaced: Toward a Development Approach” and “In My Backyard? The Economics of Refugees and Their Social Dynamics in Kakuma, Kenya” are two prime examples. The latter made the case that refugees can have positive social and economic impacts on host countries and that integration makes economic sense; while the former, “encouraged new thinking from a socioeconomic perspective” [40]. Establishing the socioeconomic benefits of integration as a policy solution was instrumental in advancing it as the newly favored policy solution.

## Discussion

Our analysis reveals that while calls for integration existed as far back as 1967, the issue only rose to prominence on the global agenda, and resulted in actual policy change, in the last decade or so. We identified several factors that facilitated the political prioritization of integration. First, the existence of a technically diverse network of humanitarian actors spanning different sectors and the ascendance of the World Bank and other development actors in the network of proponents was key. A favorable political and normative environment opened a policy window that advocates strategically leveraged. In particular, the Mediterranean refugee crisis and its securitization heightened concerns around a refugee exodus into Europe and galvanized action, resulting in the emergence of new funding instruments with unprecedented financing power. The greater movement on universal health coverage instilled a sense of urgency which proponents used to advocate for the inclusion of refugees into national health systems. Moreover, framing of integration in terms of responsibility-sharing and positioning health integration as a human right helped extend the issue beyond a health frame and facilitated the mobilization of multi-donor funds. Finally, inherent characteristics of contemporary displacement, including the severity of the refugee crisis, its increasing protractedness and urbanization, and the failings of the current paradigm made integration an appealing policy solution.

Our study highlights a number of factors which may explain why national level implementation of policies integrating refugees into health systems is not as robust as one might anticipate. For example, the Solutions Alliance which brought together diverse actors and provided an ongoing forum for discussion between global agencies, country governments and NGOs perhaps closed its doors prematurely. Ongoing interaction between these stakeholders may have helped to iron out remaining challenges in implementation. The Shiffman and Smith framework also points to the importance of a shared, coherent framing of the problem and its solutions. Our findings suggest that while there is a high degree of consensus among advocates over the key policy elements of integrating refugees into health systems, the language adopted differs (e.g. integration versus inclusion versus self-reliance). It is possible that this has facilitated uptake of the policy given sensitivities in different contexts, but as other papers in this supplement have documented, there do appear to be contested elements. For example, in contexts where access to health systems is inequitable for host populations, does integration mean aligning refugees with host populations with the most privileged access, or the least access? While global policies and mechanisms are now in place to support integration of refugees into health systems, it appears that not all national governments are supportive, and further advocacy may be needed in specific contexts. Host governments still express concern that integration will exacerbate unemployment and lead to upticks in crime, despite attempts from integration advocates to present evidence against such arguments [39, 40]. In addition, there are practical implementation challenges especially in decentralized contexts such as Uganda, where district planning and budgeting processes need to accommodate the integration of refugee populations.

Another important consideration in national level policy implementation relates to broader concerns surrounding the top-down approach to aid and international development [48]. While the integration agenda has some roots at the national level, such as the self-reliance movement in Uganda, most discourse has originated from International Organizations and high-income countries. The extent to which the top-down approach to integration might limit its implementation is yet to be fully realized but might underpin the reluctance of some national governments to adopt an integration agenda.

Perhaps one of the most significant challenges however to the full implementation of refugee integration policies is sustaining political support. It is clear that the Mediterranean refugee crisis played a major role in opening a policy window: European governments were concerned about the political cost of managing refugees who reached their own borders, and consequently were eager to support policy change in the global system and global trust funds that enhanced the prospects of refugees staying in countries of first asylum. This raises questions about the extent to which financial and technical support from the international community will materialize when crises are further from home (such as with the Rohingya refugees in Bangladesh). Further, given the protracted nature of refugee crises, host country governments are naturally concerned about the extent to which the international community will continue to support refugee integration over the longer term.

Our study should be considered in light of limitations. For one, our research question covers an extended period of time, and it may have been difficult for respondents to recall accurately events that took place more than five years ago, however a thorough document review allowed us to triangulate respondents’ recollections against documented events. Data for this study were collected in two rounds, having the benefit of allowing a more targeted second round of data collection. Finally, our study focused on chronicling events leading to the prioritization of integration as a global policy but because high level global policies seldom provide operational guidance on implementation, we were unable to speak authoritatively to implementation challenges and best practices related to integration at the national level.

## Conclusions

The Shiffman and Smith framework for political priority was an appropriate tool to analyze different factors contributing to the development of global policies and mechanisms to support the integration of refugees into health systems. Overall, at the global level, stakeholder attitudes toward integration remain positive, but on-the-ground, and as described elsewhere in this supplement, there have been challenges in sustainably integrating refugees into health systems. Our analysis identifies some of the reasons why this might be the case. In moving forward, the value of sustained advocacy should not be underestimated; this is needed both to build commitment and buy in amongst host countries that are still hesitant about the integration agenda, but also to ensure support from high income countries that is both sustained over time, and across geographies that are further afield. Further, additional work is required to translate the global agreements into practical guidance for national governments and other local stakeholders. Finally, it is vital to review the health responses to recent refugee crises, such as Ukrainian refugees in Eastern Europe, Afghan refugees in Iran and Pakistan, and Venezuelan refugees throughout Latin America and the Caribbean, to evaluate the extent to which integration polices were leveraged at the onset of these crises and to develop best practices for establishing integrated health policies and systems in the emergency phase of new humanitarian crises. While work remains to be done, the processes described in this paper have fundamentally changed the way in which refugee crises and their implications for health services are framed, and has brought about a powerful alliance between development and humanitarian actors with far reaching implications that should benefit both host and refugee populations.

### Electronic supplementary material

Below is the link to the electronic supplementary material.

Supplementary Material 1

Supplementary Material 2

## Data Availability

Data is available upon reasonable request from the corresponding author.

## Declarations

### About this supplement

This article has been published as part of Conflict and Health Volume 18 Supplement 1, 2024: Integration of Refugees into National Health Systems: Enhancing Equity and Strengthening Sustainable Health Services for All. The full contents of the supplement are available online at https://conflictandhealth.biomedcentral.com/articles/supplements/volume-18-supplement-1.
