# Supplementary material for: How integration of refugees into national health systems became a global priority: a qualitative policy analysis
Source: Confl Health. 2024 Apr 15;18(Suppl 1):31. doi: 10.1186/s13031-024-00587-4 (PMC11017473; doi:10.1186/s13031-024-00587-4)
Supplement: Supplementary file 1 — Supplementary Material 1 [file 13031_2024_587_MOESM1_ESM.docx]

**Introduction**

1. In a few sentences, could you describe the most important aspects of your organization’s work with refugees?
2. How does your organization interpret and understand “refugee integration”?
3. In your opinion, what are the top three policies and priorities for your organization regarding refugee integration and health systems?

**Refugee Integration (Policy discourse and financial priorities)**

How has your organization’s work shifted over time in terms of addressing refugee integration, particularly with respect to health services?

- 1. When do you think the approaches to refugee integration changed and why?
  2. What were the pivotal events or key factors that might have caused this shift?
  3. Who or which organizations or governments do you believe had central roles in driving these policy changes?
  4. How would you characterize the role of your organization in these policy shifts?

1. How have donor funding commitments and priorities changed over time for refugee integration?
   1. Why do you believe some areas have been prioritized over others?
   2. How were funding commitments adjusted to account for changes in policy and who were the key stakeholders driving this process?
   3. Were the main drivers the mandates of global level actors, governments, or are there other factors causing this change?
2. What are the challenges that your organization has encountered in terms of supporting refugee integration into health services?
   1. Can you provide any ‘positive’ examples and what factors caused them to succeed?
   2. Can you provide any ‘challenging’ examples and what factors caused them not to succeed?

Global/National Policy Shifts and Org

1. Can you think of any cases where these global level policy shifts may have shaped national level health policies?
2. Can you think of cases where national level dynamics have shaped global level policy shifts?
3. To what extent do you feel that there are coherent policies across this organization on refugee integration?
   1. Can you tell me more about how your team within this organization communicates with other teams working on these issues?

Health System Access: Priorities and Development

1. How do you believe that changing policies on refugee integration in health systems have affected health service provision?
   1. How does this vary for different types of services (e.g. primary care, versus referral services; notifiable infectious diseases versus others)?
   2. How do you think this differs for refugee and host populations?
   3. How do you believe these policy developments have affected front line healthcare providers?
2. What are some of the support systems that your organization and other development partners have put in place to support refugee integration into health systems?
3. Refugees may have unique needs from health systems- for example, mental health and SGBV, can you expand on how these factors have influenced the type of support that you might provide to a country health system?
   1. How is provision of services and allocation of resources prioritized? [e.g., acute or vulnerable populations, MNCH, HIV, victims of SGBV, special needs and disabilities]
4. There are some documents that mention how providing refugees with access to national health services will also bring benefits to host populations—do you believe that is the case?
   1. How have national policymakers reacted to this?
   2. How do you make the case for it when this may be seen as an additional burden on health systems?
5. Health workforce policies for refugee health workers varies among countries—how has your organization addressed the barriers that refugee health workers face?

**Additional Comments**

- Follow up on any documents or key policies mentioned in the interview
- Is there anything you would like to share that I may not have touched upon?
- Ask if there are additional people they think it would be good to talk to.
