# Supplementary figures and images for: How integration of refugees into national health systems became a global priority: a qualitative policy analysis
Source: Confl Health. 2024 Apr 15;18(Suppl 1):31. doi: 10.1186/s13031-024-00587-4 (PMC11017473; doi:10.1186/s13031-024-00587-4)

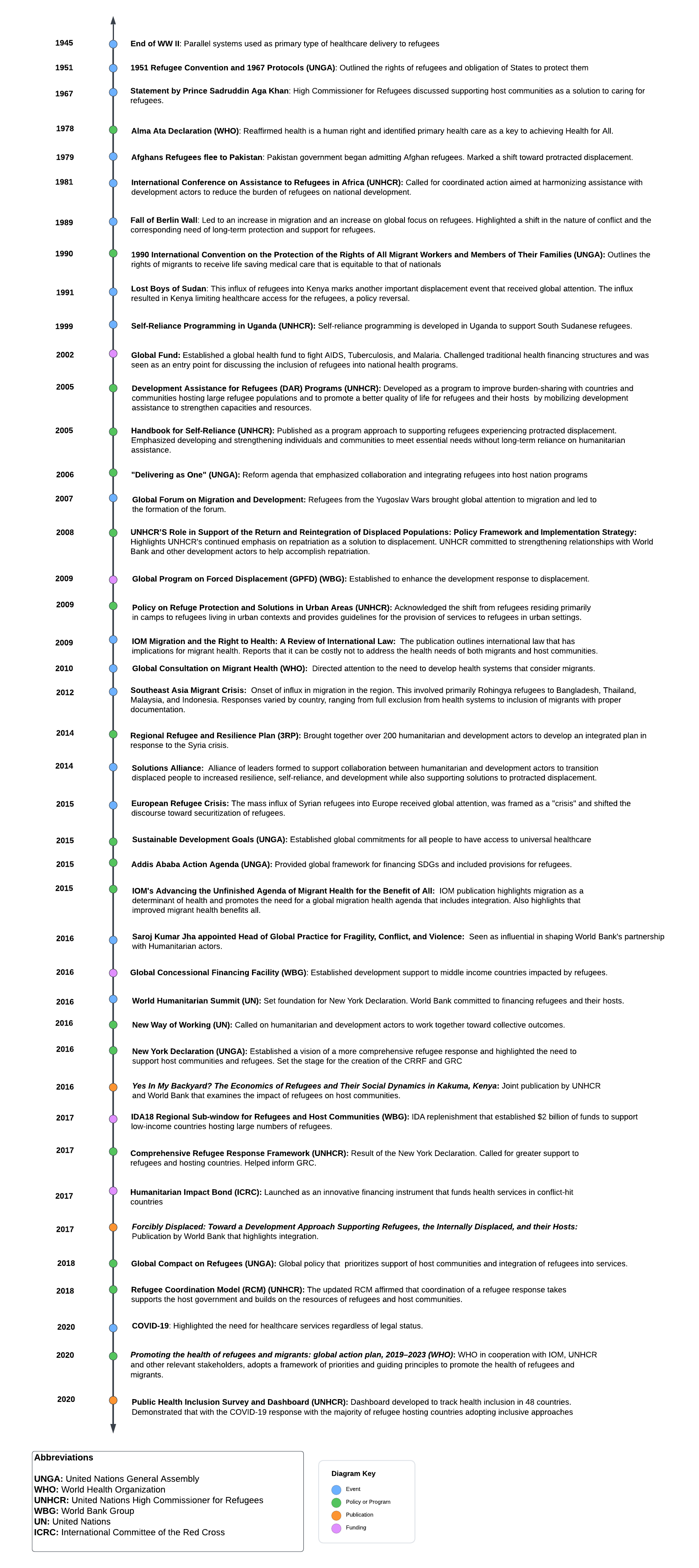

Supplement: Supplementary file 2 — Supplementary Material 2 [file 13031_2024_587_MOESM2_ESM.png]
